# Variation in Plumage Coloration of Rosy‐Faced Lovebirds (*Agapornis roseicollis*): Links to Sex, Age, Nutritional Condition, Viral Infection, and Habitat Urbanization

**DOI:** 10.1002/jez.2867

**Published:** 2024-09-16

**Authors:** Kevin J. McGraw, Reilly Hammond, Simona Kraberger, Arvind Varsani

**Affiliations:** ^1^ School of Life Sciences Arizona State University Tempe Arizona USA; ^2^ The Biodesign Center for Fundamental and Applied Microbiomics Arizona State University Tempe Arizona USA

**Keywords:** *Circovirus parrot*, condition‐dependent signaling, *Gammapolyomavirus avis*, parrots, sexual dichromatism

## Abstract

Expression of vibrant plumage color plays important communication roles in many avian clades, ranging from penguins to passerines, but comparatively less is known about color signals in parrots (order Psittaciformes). We measured variation in coloration from three plumage patches (red face, blue rump, red tail) in an introduced population of rosy‐faced lovebirds (*Agapornis roseicollis*) in Phoenix, Arizona, USA and examined color differences between the sexes and ages as well as relationships with several indices of quality, including disease presence/absence (infection with beak and feather disease, *Circovirus parrot*, and a polyomavirus, *Gammapolyomavirus avis*), nutritional state (e.g., blood glucose and ketone levels), and habitat type from which birds were captured. We found that different plumage colors were linked to different quality indices: (a) adults had redder faces than juveniles, and birds with brighter faces had lower glucose levels and were less likely to have polyomavirus; (b) males had bluer rumps than females; and (c) birds caught farther from the city had redder and darker tail feathers than those caught closer to the urban center. Our findings reveal diverse information underlying variation in the expression of these disparate, ornate feather traits in an introduced parrot species, and suggest that these condition‐dependent and/or sexually dichromatic features may serve important intraspecific signaling roles (i.e., mediating rival competitions or mate choices).

## Research Highlights

- Lovebird adults had redder faces than juveniles, and birds with brighter faces had lower glucose levels and were less likely to have polyomavirus.
- Male lovebirds had bluer rumps than females.
- Lovebirds caught farther from the city had redder and darker feathers than those caught closer to the urban center.

## Graphical abstract

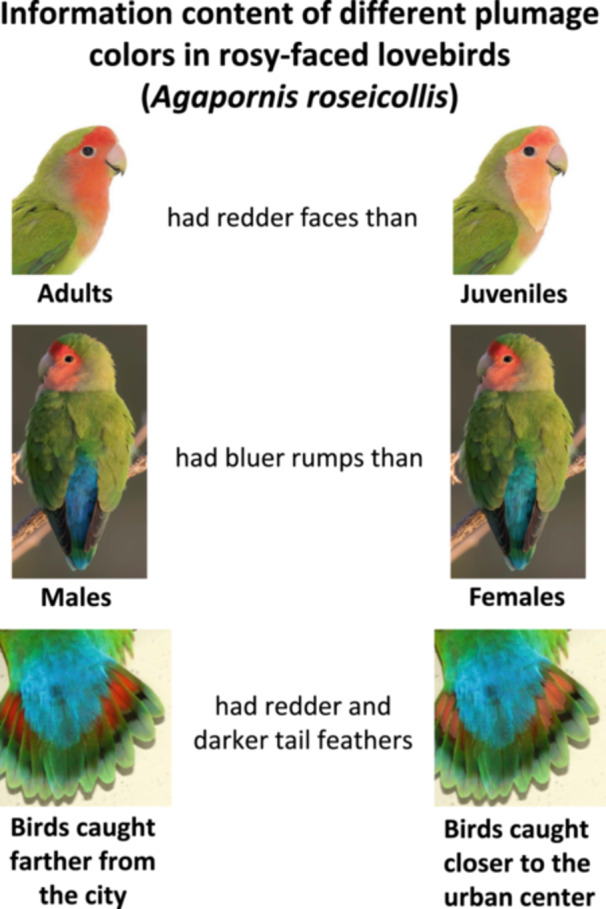

## 1 Introduction

Many birds have brightly colored plumage, which serves key visual‐signaling roles either within (e.g., social status, mate attractiveness; Soma and Garamszegi 2018; Mason and Bowie 2020) or among species (e.g., species recognition; anti‐predator or ‐parasite; McNaught and Owens 2002; Caro and Allen 2017). Intraspecific color signals in particular can be sexually dichromatic, highly variable among individuals (in the ornamented sex(es)), and carry differential production costs that permit birds to honestly reveal their quality to rivals or potential mates (Carballo et al. 2020; Delhey et al. 2023). To date, the regulation and function of sexually dichromatic, condition‐dependent plumage traits have been well‐studied in several avian clades, ranging from penguins (Sphenisciformes; Cairns 1986; Massaro, Davis, and Darby 2003; Viera et al. 2008) to pigeons (Columbiformes; Mahler, Araujo, and Tubaro 2003; Valdez and Benitez‐Vieyra 2016; Angelier 2020) to passerines (Passeriformes; McQueen et al. 2019; Cooney et al. 2022; Thibault et al. 2022).

Despite being speciose (ca. 400 species worldwide) and one of the most wildly colorful avian groups, we know comparatively less about the controls and roles of conspicuous coloration in parrots (Order Psittaciformes). Parrots produce the full rainbow of colors in their plumage, even within a species (rainbow lorikeet, *Trichoglossus moluccanus*), yet only in a handful of comparative studies and even fewer intraspecific studies have behavioral ecologists probed the mechanisms and meanings of ornate parrot colors (Berg and Bennett 2010; Delhey and Peters 2017). For example, color diversity among parrot species can be explained by the ambient climate and body size, such that smaller species are more sexually dichromatic but that larger species living in warmer environments tend to be more colorful overall (Carballo et al. 2020). Perhaps related to climatic pressures, more colorful parrot feathers are able to better resist bacterial degradation (Burtt et al. 2011). Within species, scientists have sought to understand color variation and signaling roles in model species like the budgerigar (*Melopsittacus undulatus*, Pearn, Bennett, and Cuthill 2001) and crimson rosella (*Platycercus elegans*, Berg et al. 2019), but several of these investigations are on captive or pet birds (also see van der Zwan, Visser, and van der Sluis 2019), with exceptions like the burrowing parrot (*Cyanoliseus patagonus*, Masello et al. 2004; Masello, Lubjuhn, and Quillfeldt 2008) and eclectus parrot (*Eclectus roratus*; Heinsohn 2008) being subjects for studies of natural plumage variation and function. Overall, we are in need of more single‐species investigations into the variability and predictors of color expression in free‐ranging parrots, to better understand how these visual traits—among the most exaggerated in the animal kingdom—evolve and function.

**Figure 1:**
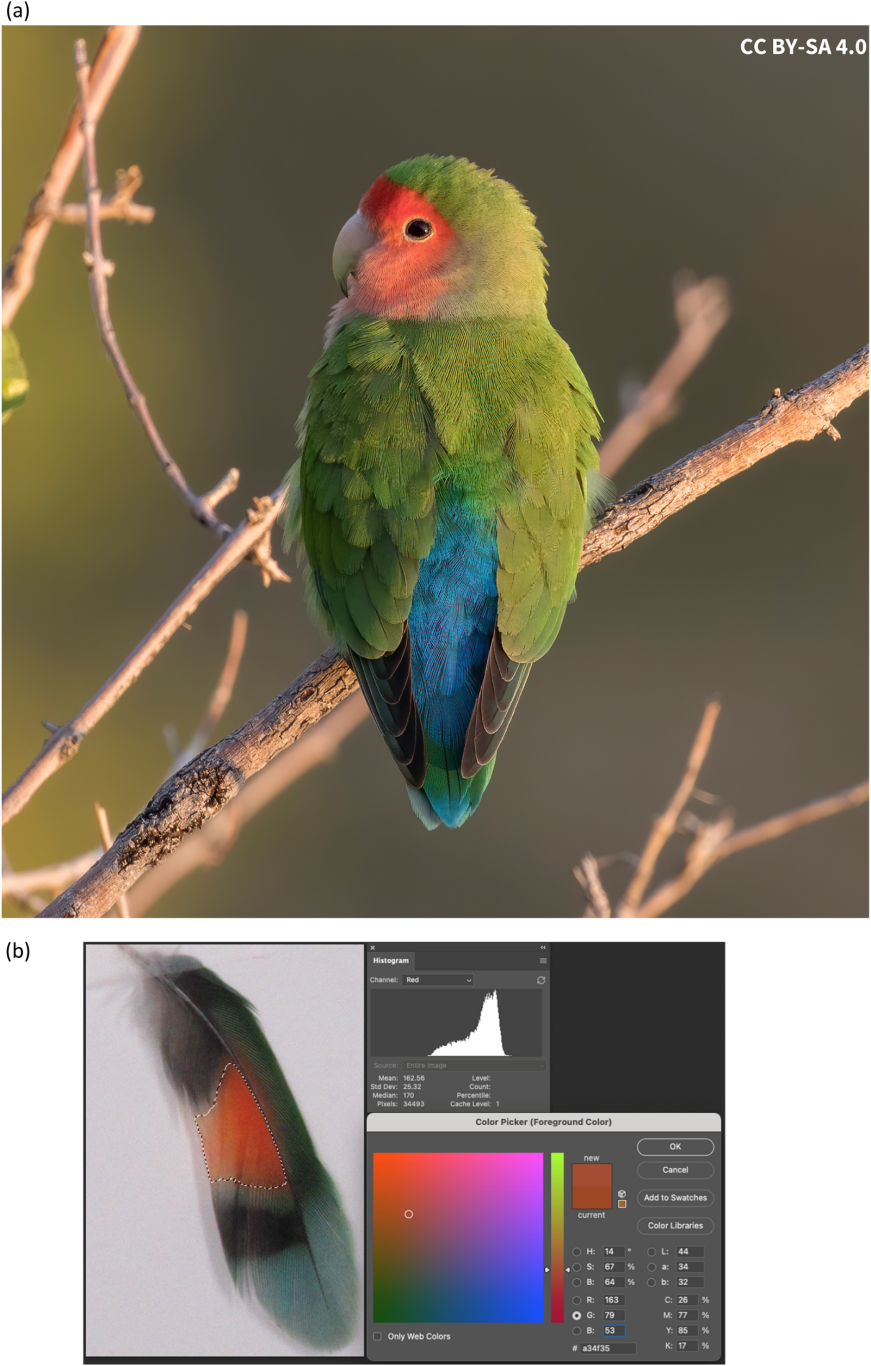
(a) Image of a rosy‐faced lovebird (*Agapornis roseicollis*), exhibiting its peach face and blue rump coloration (https://upload.wikimedia.org/wikipedia/commons/b/bf/Rosy-faced_lovebird_%28Agapornis_roseicollis_roseicollis%29_2.jpg). (b) Screenshot from color calculations made with Adobe Photoshop, including image of red tail spot. The lasso marquee was used to select the red area, and then the Histogram function provided red/green/blue (RGB) values (for calculating hue/saturation/brightness [HSB] using the Color Picker function) as well as pixel count, which was used to calculate tail spot area in mm^2^ using a size standard (ruler) included in each photo.

Here, we undertook a field investigation into the variation in and predictors of bold plumage color expression in rosy‐faced lovebirds (*Agapornis roseicollis*). This species is native to the dry regions of southwestern Africa (e.g., Angola, Namibia, South Africa; Collar and Boesman 2020) and well‐known internationally in the pet trade (Chan et al. 2021), as well as for its pair‐bonding behavior and range of plumage variations and mutants (van der Zwan et al., 2018). Birds of both sexes display conspicuous rosy‐colored feathers on the face, a brilliant blue rump patch, and spots of red pigmentation on otherwise dark tail feathers (Figure 1). Early spectral‐reflectance studies were performed to characterize the reflectance and structures of green and blue plumage patches in this species (Dyck 1971; also see Tinbergen, Wilts, and Stavenga 2013 in Amazon parrots), but interindividual studies of natural color variation, including for the unique psittacofulvin‐based red pigmentation (to parrots; McGraw and Nogare 2005) after which these birds are named, are lacking. Specifically we studied an introduced population of lovebirds in North America (Phoenix, Arizona, USA), which is derived from a pet‐bird release nearly 40 years ago and has radiated throughout the built metropolitan area, likely due to human‐provided access to water in this desert city (Radamaker and Corman 2011).

We quantified color variability for the three aforementioned plumage patches in male and female lovebirds, including both juveniles and adults, to determine if there is age‐ or sex‐specific expression of color (e.g., as in yellow‐faced parrots, *Alipiopsitta xanthops*; de Araújo and Marcondes‐Machado 2014). We also captured birds at different sites across the urban–suburban landscape of Phoenix, Arizona, USA, to evaluate the hypothesis that cities and associated human activities can impact bird color expression (Leveau 2021), but under the notion that few tests of this (i.e., in house sparrows, *Passer domesticus*; rock pigeons, *Columba livia*) have been done in introduced species. We also measured a series of quality‐related indices in these birds, to determine if plumage color variation may reveal an individual's health or condition; specifically, we considered nutritional status (e.g., body condition, blood glucose, and ketone levels; DePinto and McGraw 2022), given prior work on blood indices and color expression in burrowing parrots (Masello and Quillfeldt 2004), and disease status (e.g., viral infection). Parrots are host to several viral infections, including beak‐and‐feather‐disease virus (BFDV—*Circovirus parrot*; Fogell, Martin, and Groombridge 2016) and a polyomavirus (budgerigar fledgling disease virus, BuFDV—*Gammapolyomavirus avis*; Padzil, Mariatulqabtiah, and Abu 2017) in this species (Ko et al. 2024); we molecularly characterized these infections in these birds to determine if, as has been shown in other birds, more colorful birds are less likely to be infected (Hill and Farmer 2005). Because this was an exploratory study, we did not have specific predictions about how coloration of the different plumage regions may similarly or differently relate to our predictor variables.

## 2 Methods

### 2.1 Field Capture and Sampling

From 2 to 13 June 2022, we live‐trapped a total of 69 lovebirds (showing wild‐type plumage) using hanging basket traps at baited feeding stations at four sites within the metropolitan area of Phoenix, AZ, USA: (1) Cholla Park (Scottsdale, AZ; 33.589550, −111.838687), (2) the Desert Arboretum Park at Arizona State University (Tempe, AZ; 33.426126, −111.930185); (3) Encanto Park (Phoenix, AZ; 33.475516, −112.090166), and (4) Arizona Fruit Trees Nursery (Mesa, AZ; 33.413422, −111.651620)—which varied in natural v. artificial habitat characteristics and distance to city center (Supporting Information S1: Figure 1). At capture, we determined age (juvenile vs. adult) of each bird based on bill pigmentation (black in juveniles; Ndithia, Perrin, and Waltert 2007) and measured body mass (to the nearest 0.1 g with a digital scale) and tarsus length (to the nearest 0.1 mm with digital calipers) to calculate body condition (i.e., residual mass, from a linear tarsus‐mass regression). We drew blood from the alar vein to molecularly determine sex (using primers 2550/2718; *sensu* Fridolfsson and Ellegren 1999) of each bird and for analyses of nutritional condition and viral disease status (see more below). As indices of nutritional condition, we measured glucose and ketone levels from a drop of fresh blood using hand‐hand point‐of‐care devices (DePinto and McGraw 2022).

### 2.2 Virus Analyses

At capture, we also used sterile swabs to gently swab the cloaca of each bird, which was preserved in UTM Universal Transport Media (Copan, USA). Viral DNA was extracted from the UTM media by using 200 µL of the buffer and the High Pure Viral Nucleic Acid Kit (Roche Diagnostics, USA). To enrich for circular DNA, 1 μL of extracted viral DNA from each sample was used for rolling‐circle amplification (RCA) using a TempliPhi Kit (GE Healthcare, USA). One microliter of the RCA was used as a template with Kapa HiFi polymerase (Roche, USA) in a polymerase chain reaction with primers BFDV_F CGCGCGAGAGTTCCCASA and BFDV_R ACTTCCTTCATTTTRCRTCCGG to screen for BFDV (*Circovirus parrot*) and BuFDV F TGTCGTCGTTGATCGTGGGGAGC and BuFDV R TTACGTGCCCGACCCTGCTTATGTG to screen for BuFDV (*Gammapolyomavirus avis*). Cycling conditions were applied in accordance with the manufacturer recommended protocol. Forty six of the 69 lovebirds (67%) tested positive for BFDV, whereas six birds (9%) tested positive for BuFDV.

### 2.3 Color Measurement

We used digital photography (Stevens et al. 2007; Troscianko and Stevens 2015)—with standard illumination conditions (camera flash), distance from camera to object (25 cm), background surface (Kodak R‐27 gray card), and color standard with ruler (Kodak Color Control Patches; also see additional details in Giraudeau et al. 2013; Hutton, McKenna, and McGraw 2021)—to quantify color and patch‐size variation (for tail spots) in the lovebirds. We plucked 8–10 feathers from a central location of the rosy face and blue rump patches, as well as the outermost right tail feather (containing a red spot), and mounted these on cardstock for imaging. We did not measure green feathers because this is the base body color of these, and many other species, of parrots. RAW photos of these feather cards were analyzed in Adobe Photoshop (McGraw, Lee, and Lewin 2011) to determine hue, saturation, and brightness of the face, rump, and tail feathers, as well as the size of the red tail spot. We acknowledge that this photographic analysis omits ultraviolet (UV) reflectance (Dyck 1971; Zhang et al. 2014; though it is noteworthy that UV reflectance is very low in red‐colored parrot feathers; Burkhardt 1989, McGraw and Nogare 2005), but in this first study of its kind in this species, we characterize the visible‐light spectral properties (as in Masello et al. 2004 in burrowing parrots) and capitalize on the opportunity, unlike with spectrometry, to quantify tail spot size. Two independent observers scored each photo and we found significant, positive repeatability of all 10 scores (face hue = 0.95, face saturation = 0.79, face brightness = 0.96, rump hue = 0.92, rump saturation = 0.70, rump brightness = 0.59, tail‐spot hue = 0.94, tail‐spot saturation = 0.85, tail‐spot brightness = 0.96, tail‐spot size = 0.93; all *p* < 0.001; Lessells and Boag 1987); mean values were used for each color metric below in statistical analyses.

### 2.4 Statistical Analyses

First, to examine potential multicollinearity among response variables, we examined intercorrelations among our 10 colorimetrics and found significant correlations between hue and saturation for all three color patches, and between several other tail variables (saturation and brightness, saturation and spot size, and brightness and spot size; Supporting Information S2: Table 1). Thus, we retained only hue and brightness scores for the three patches in our plumage‐color analyses (i.e., six response variables total). Lower hue scores for the red face and tail correspond to redder (less orange) colors, whereas lower hues for rump feathers signify a more green (less blue) appearance; in all cases for saturation and brightness, respectively, higher saturation values correspond to purer (more rich) color and higher brightness values indicate lighter (less dark) color. We also examined relationships among independent variables (age, sex, capture site, body condition, ketones, glucose, BFDV presence, and BuDFV presence) and found only that body condition was significantly negatively related to blood ketone levels (Supporting Information S2: Table 2); given our aim to use more refined metrics of nutritional‐physiological condition in this study, we retained only blood ketone levels and omitted body condition from further analyses.

We used JMP Pro 16 (SAS Institute Inc., Cary, NC) to construct standard least‐square regression models predicting plumage color variation. We entered all predictors and report global models, but also used Akaike's Information‐Theoretic Criterion (AICc) on all possible models (predictor subsets/combinations) to select the best‐fit model, based on the lowest AICc score.

## 3 Results

### 3.1 Face Patch

**Figure 2:**
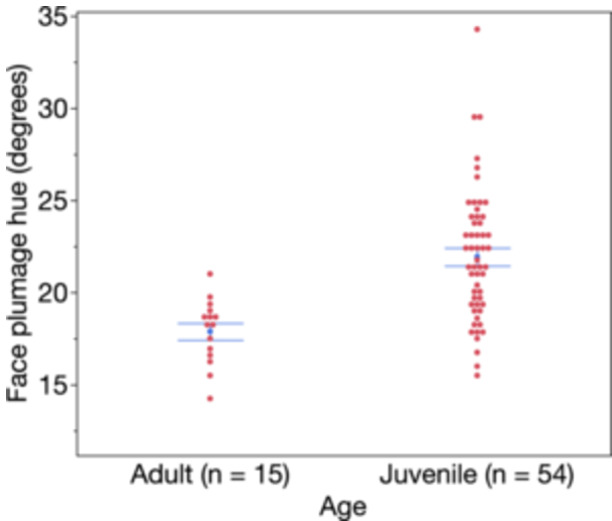
Effect of age (adult vs. juvenile) on the hue of face plumage in rosy‐faced lovebirds. Mean ± SE is shown in blue, plus individual (red) points horizontally offset so as not to overlap. Lower hue scores denote birds with redder (less orange) faces.

**Figure 3:**
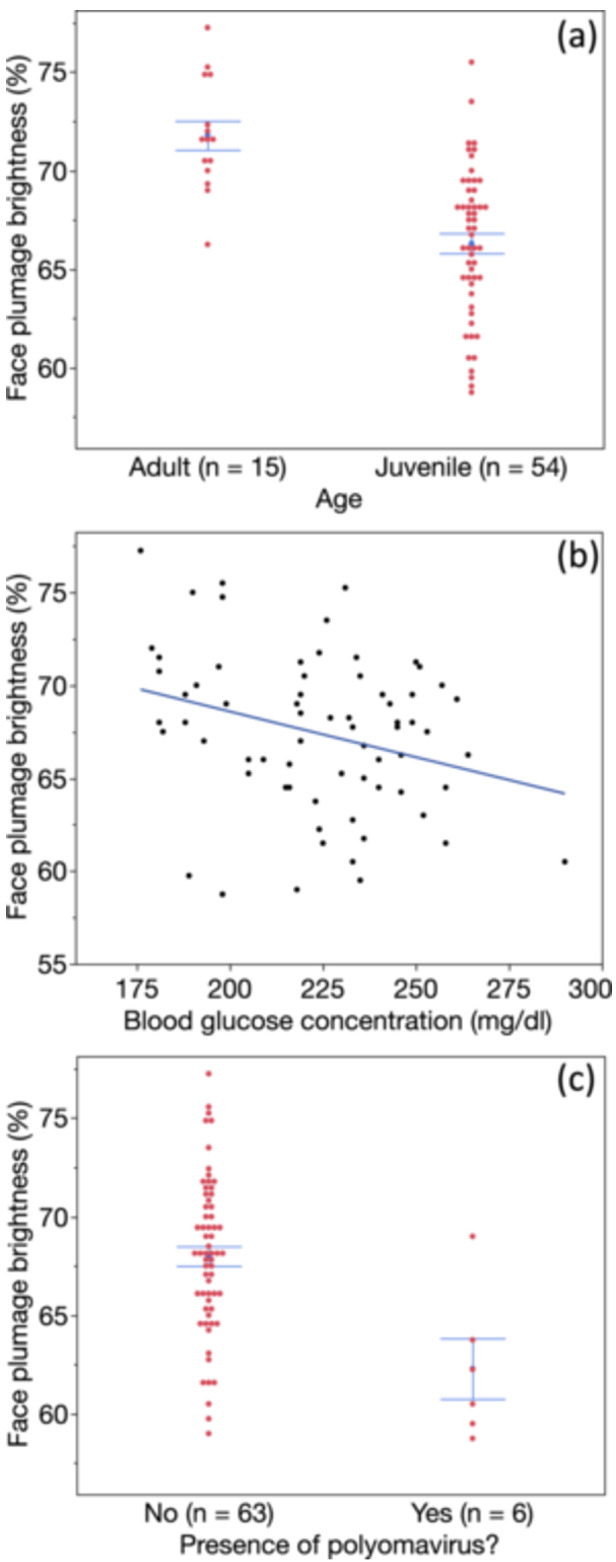
Links between brightness of facial plumage and (a) age, (b) circulating glucose levels, and (c) presence of budgerigar fledgling disease virus (BuFDV). Adults were brighter than juveniles, and birds with less bright (darker) plumage circulated more glucose and were more likely to be infected with polyomavirus.

The global model predicting hue of face feathers yielded only one significant predictor, age (Table 1), such that older birds had redder face feathers (Figure 2). Age was the lone, retained variable (still statistically significant) predictor in the best‐fit model (Table 1). In contrast, for brightness of face feathers, we found in the global model that blood glucose levels, presence of BuFDV, and age were significant predictors (Table 1), and the best‐fit model predicting face brightness contained only these three parameters (Table 1), such that birds with brighter face feathers were older, had lower glucose levels, and were less likely to have BuFDV (Figure 3).

**Table 1:** Results of models predicting variation in facial plumage hue and brightness in rosy‐faced lovebirds.

Trait	Model	Parameter	Estimate	*F*	*p*
(a) Face hue	Global	Sex	−0.53	1.61	0.21
*r* ^2^ = 0.29		**Age**	**−2.24**	**18.70**	**< 0.0001**
		Site	—	1.07	0.37
		Blood glucose levels	0.01	0.25	0.62
		Blood ketone levels	−0.15	0.25	0.62
		BFDV presence	0.33	0.46	0.50
		BuFDV presence	0.62	0.63	0.43
	Best‐fit	**Age**	**−2.02**	**17.78**	**< 0.0001**
(b) Face brightness	Global	Sex	−0.07	0.03	0.87
*r* ^2^ = 0.47		**Age**	**2.49**	**23.38**	**< 0.0001**
		Site	—	0.00	1.00
		**Blood glucose levels**	**−0.04**	**5.32**	**0.02**
		Blood ketone levels	0.04	0.02	0.88
		BFDV presence	−0.08	0.03	0.87
		**BuFDV presence**	**2.80**	**13.11**	**0.001**
	Best‐fit	**Age**	**2.48**	**26.97**	**< 0.0001**
		**Blood glucose levels**	**−0.04**	**7.27**	**0.009**
		**BuFDV presence**	**2.82**	**17.52**	**< 0.0001**

*Note:* Parameters in bold were statistically significant.

### 3.2 Rump Patch

**Figure 4:**
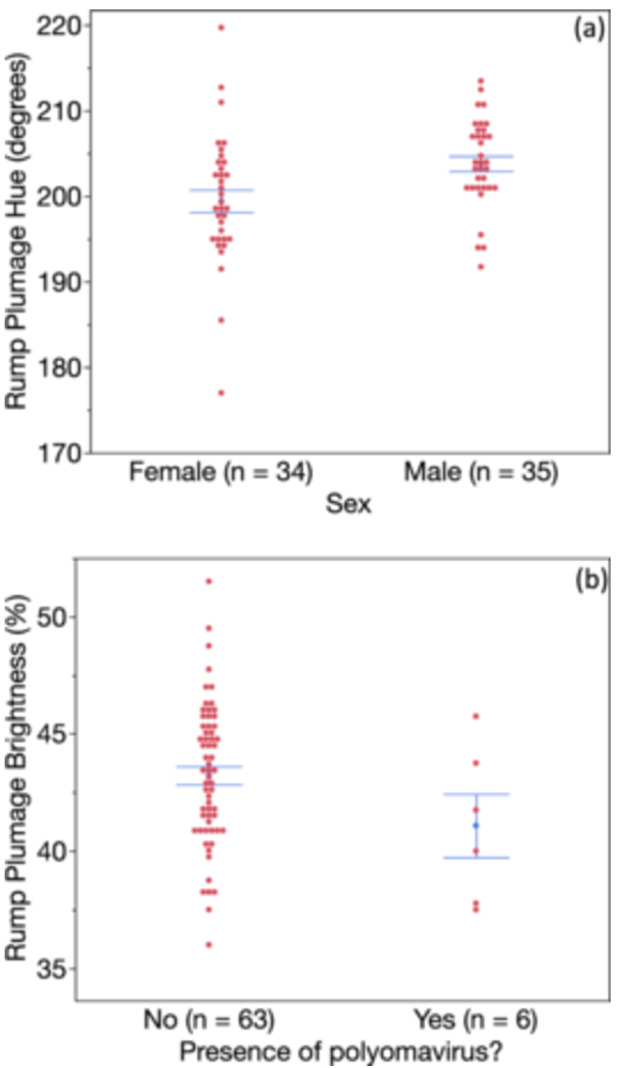
Variation in rump plumage coloration as a function of sex and presence of a polyomavirus (BuFDV, *Gammapolyomavirus avis*) in rosy‐faced lovebirds. Higher hue scores denote birds with bluer (less green) rumps. Males had bluer rump plumage compared to females, and birds with darker rumps were more likely to have the BuFD polyomavirus.

The global model predicting rump hue yielded two significant predictors—sex and presence/absence of BFDV (Table 2). These two variables were retained in the best‐fit model as well, though only sex was statistically significant (Table 2), such that rump feathers from males were bluer (less green) than those of females (Figure 4a). In contrast, BuFDV infection presence predicted rump brightness, both as the lone significant factor in the global model and the lone factor (though not statistically significant) in the best‐fit model (Table 2); birds with brighter rumps were less likely to be infected (Figure 4b).

**Table 2:** Results of models predicting variation in rump plumage hue and brightness in rosy‐faced lovebirds.

Trait	Model	Parameter	Estimate	*F*	*p*
(a) Rump hue	Global	**Sex**	**−2.10**	**6.66**	**0.01**
*r* ^2^ = 0.23		Age	0.48	0.22	0.64
		Site	—		
		Blood glucose levels	0.01	0.07	0.80
		Blood ketone levels	−0.25	0.20	0.66
		**BFDV presence**	**−2.08**	**4.79**	**0.03**
		BuFDV presence	0.88	0.34	0.56
	Best‐fit	**Sex**	**−2.15**	**7.58**	**0.008**
		BFDV presence	−1.38	2.68	0.11
(b) Rump brightness	Global	Sex	0.24	0.37	0.54
*r* ^2^ = 0.15		Age	0.39	0.65	0.42
		Site	—	1.68	0.18
		Blood glucose levels	−0.01	0.54	0.47
		Blood ketone levels	−0.10	0.14	0.71
		BFDV presence	0.49	1.17	0.28
		**BuFDV presence**	**1.53**	**4.56**	**0.04**
	Best‐fit	BuFDV presence	1.07	2.70	0.11

*Note:* Parameters in bold were statistically significant.

### 3.3 Tail Spot

**Figure 5:**
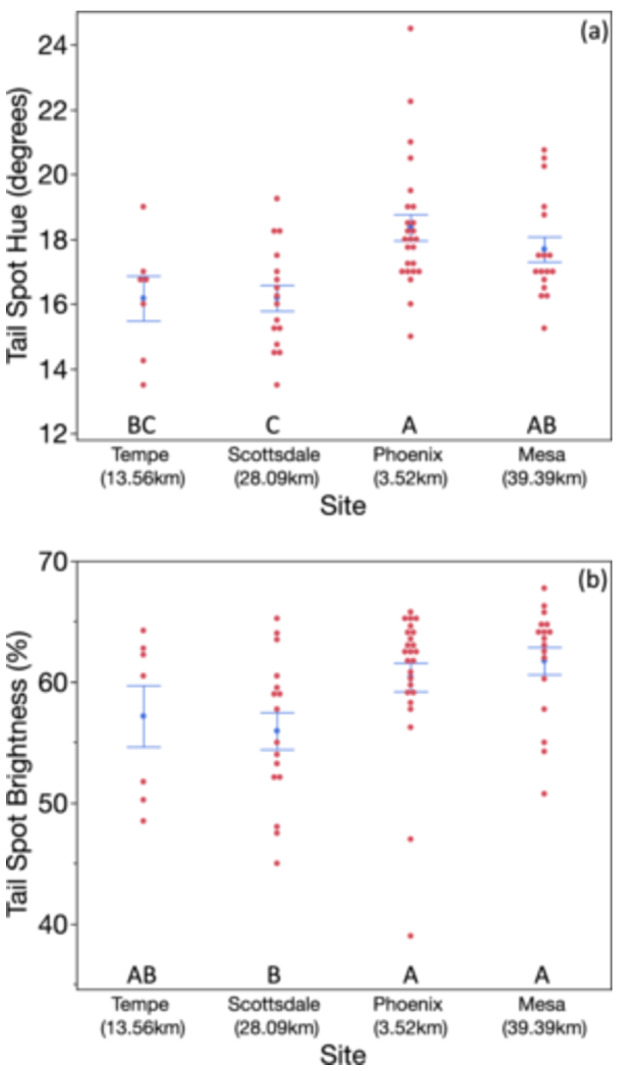
(a, b) Effects of capture site on tail spot hue and brightness in rosy‐faced lovebirds. Birds with lower hue scores had redder tail spots. Unshared letters denote statistically significant differences between groups. In the *x*‐axis labels, km stands for distance in kilometers to city center. We found that lovebirds from the most urban site (Encanto Park, Phoenix) had the least red tail spots and that birds from the most rural park (Scottsdale) had the darkest tail spots.

Capture site predicted both tail spot hue and brightness, such that lovebirds from the most urban site (i.e., closest to city center = Encanto Park, Phoenix) had the least red tail spots and that birds from the most rural park (i.e., further from city center = Scottsdale) had the darkest tail spots (Table 3, Figure 5).

**Table 3:** Results of models predicting variation in tail spot hue and brightness in rosy‐faced lovebirds. Parameters in bold were statistically significant.

Trait	Model	Parameter	Estimate	*F*	*p*
(a) Tail hue	Global	Sex	0.08	0.12	0.73
*r* ^2^ = 0.28		Age	0.37	1.60	0.21
		**Site**	**—**	**3.83**	**0.01**
		Blood glucose levels	0.00	0.00	0.99
		Blood ketone levels	0.07	0.17	0.68
		BFDV presence	0.29	1.08	0.30
		BuFDV presence	−0.21	0.24	0.63
	Best‐fit	**Site**	**—**	**6.05**	**0.001**
(b) Tail brightness	Global	Sex	1.09	2.18	0.15
*r* ^2^ = 0.23		Age	−0.18	0.04	0.84
		**Site**	**—**	**3.03**	**0.04**
		Blood glucose levels	0.01	0.03	0.85
		Blood ketone levels	−0.37	0.52	0.47
		BFDV presence	0.84	0.93	0.34
		BuFDV presence	−1.81	1.81	0.18
	Best‐fit	**Site**	**—**	**3.45**	**0.02**

## 4 Discussion

We examined several life‐history, environmental, and physiological predictors of plumage color variation in an introduced population of rosy‐faced lovebirds and found that coloration from different plumage regions (face, rump, tail) covaried with different variables. Expression of facial plumage was age‐dependent and also linked to a nutritional measure (glucose) and to disease status (BuFDV infection). In contrast, rump plumage color was sex‐specific but also varied as a function of infection with both viruses (BFDV and BuFDV). Last, coloration of the tail spot was predicted only by capture site (i.e., distance to city center). To our knowledge, this study is one of few that has extensively examined complex (i.e., multiple patch) plumage color variation in a wild parrot (Berg and Bennett 2010; also see Masello, Lubjuhn, and Quillfeldt 2008 in burrowing parrots).

Age‐dependence of red facial plumage in *A. roseicollis* was an expected finding; from observations of captive/pet birds, juvenile rosy‐faced lovebirds, as in many other parrots, are known to show muted (i.e., less red) facial coloration compared to adults (Forshaw 2010). This is largely thought to link to sexual maturity and adult expression of secondary sexual characters, although there may be added camouflage advantages for naïve juveniles to be duller (Mitchell 2019). We also found that lovebirds with brighter facial feathers circulated a lower concentration of glucose through blood and were less likely to have BuFDV. Glucose can be a reliable, positive biomarker of (carbohydrate) energy status and, though we lack comparable data for parrot plumage, other avian work has revealed the absence of a relationship between glucose and plumage coloration (e.g., carotenoid pigmentation in finches, McGraw et al. 2020; melanin coloration in gulls, Minias et al. 2019). Glucose can also be linked to disease status in humans (e.g., diabetes, obesity; Chen et al. 2019) and birds (e.g., higher levels in poxvirus‐infected finches, McGraw et al. 2020), and interestingly we also found that lovebirds with brighter face feathers were also less likely to be infected by a contagious virus (polyomavirus) that is common to psittacines and can impact feather condition and even become fatal in some cases (Katoh et al. 2010; Dolz et al. 2013). On one hand, the most parsimonious explanation for our detected association between face brightness and BuFDV infection may be the direct impact of the virus on the structure (e.g., degradation; Johne and Müller 2007) of small/fine facial feathers, but in future work, it will be interesting to examine integrated physiological (perhaps glucose‐mediated) underpinnings of both this viral infection and facial plumage development/maintenance.

In addition to red pigmentary coloration, we explored several possible correlates of structurally based blue rump color expression in these birds. We detected significant sexual dichromatism in rump hue, with males having bluer (less green) rumps than females. Sex differences in structural blue plumages has been shown previously in other parrot species (e.g., blue‐fronted Amazon parrot (*Amazona aestiva)*, Santos, Elward, and Lumeij 2006; remiges in burrowing parrots, Masello, Lubjuhn, and Quillfeldt 2009), and intriguingly, in a comparative study of 27 Australasian parrot species, sexual plumage dichromatism was strongest and most consistent for blue colors (Taysom, Stuart‐Fox, and Cardoso 2011; but see Delhey and Peters 2017 for an analysis that shows comparable dichromatism between parrot structural and psittacofulvin plumage coloration). Potentially this conspicuous blue plumage (Figure 1a) in rosy‐faced lovebirds may serve as a strong target for selection as a visual signal (e.g., intrasexual competition or intersexual mate choice), especially in males (where it's bluer) and additionally given the fact that we found, like for facial plumage, a link between BuFDV prevalence and blue plumage brightness (although this was not significant in the best‐fit model). The presence of brighter feathers by BuFDV‐free birds in two different plumage regions suggests a robust effect of this virus on the ornate feathers in this species (i.e., impaired structural integrity in two regions, which are based on different underlying color mechanisms) and notably is the lone predictor for which we found consistent effects across plumage regions. In future experimental work, it will be interesting to see if lovebirds gain useful information about disease status by assessing a competitor's or potential mate's blue plumage color variability.

Last we examined indicators of condition dependence in a “hidden plumage trait” in these birds—the red spot on tail feathers that is not visible in perched lovebirds, and only when they perform courtship displays and present a fanned‐out tail to prospective mates (Spoon 2006). At first glance, given the shared red color and psittacofulvin‐based pigmentary mechanism underlying the facial and tail‐spot plumages, we may have expected to see coloration of these two patches to relate similarly to sex, age, habitat, and/or condition. However, we did not find any significant correlations between facial and tail plumage‐color metrics (Supporting Information S2: Table 1), and we also found different predictors of red tail color from those of facial pigmentation. Interestingly, birds with more heavily pigmented tail spots (i.e., darker, redder) were found at more rural areas. Prior work has shown effects of urbanization on several different types of avian plumage colors (carotenoid: Jones, Rodewald, and Shustack 2010; Baldassarre et al. 2023; melanin: Csanády and Duranková 2021; structural: Yeh 2004), but we believe our findings are the first to uncover such a relationship for psittacofulvin‐based plumage coloration. In such studies, it has been proposed that city‐related shifts in diet or stress (e.g., oxidative, immune) in the birds could mechanistically shape “urban dullness” (Leveau 2021); it is also possible that functional variation—that is, in the value or use of the signal in the city—could contribute to the observed pattern, such that urban‐associated lighting conditions or the socioecological pressures for competition or mating could lead to a reduced investment by the birds in (or value of) the hidden red plumage trait (Hutton and McGraw 2016). However, we note that we did not carefully quantify degree of urbanization in this study, so this result must be interpreted with caution. We also suggest future work to probe how and why “urban dullness” may persist for this unique plumage trait, and not other feather regions in this species, including one that shares a similar pigmentary basis.

In summary, by uncovering unique life‐history/environmental/physiological predictors of color expression across three ornate plumage regions in an introduced, city‐dwelling population of rosy‐faced lovebirds in the United States, we found support for our hypotheses that variation in feather coloration can link to important individual (e.g., sex, age) and environmental (e.g., urbanization) traits in this species. It will be interesting now to expand this work to include more birds (i.e., beyond our limited sample of six adults) at different times of year and in different parts of their introduced range (i.e., Hawaii), as well as to consider whether these results apply to native populations of *A. roseicollis* in southwestern Africa as well as other *Agapornis* and parrot species with these blue and red colors. Nevertheless, our results here suggest that these birds have the opportunity to signal different aspects of their health/condition/status with their facial, rump, and tail ornaments, and that these same traits may be very useful to biologists interested in tracking the real‐time impacts of rapidly changing environmental conditions—such as urbanization and disease spread—on wildlife (Hill 1995).

## Author Contributions

Kevin J. McGraw designed the study, led the student‐assisted field work and sample analyses, and wrote the manuscript. Reilly Hammond contributed to field work and analyses, and offered comments on the manuscript. Simona Kraberger and Arvind Varsani led the viral analyses and contributed to manuscript writing and editing.

## Ethics Statement

These birds were studied under approval of the Institutional Animal Care and Use Committee at Arizona State University (protocol # 21‐1833R).

## Conflicts of Interest

The authors declare no conflicts of interest.

## Supporting information


**Supplemental Figure 1** Inset map (expanding local area from the red star demarcating the greater Phoenix, Arizona, USA metropolitan area) and satellite images of the 4 numbered study sites. (1) Encanto Park—developed park with artificial lakes, playground, walking paths, amusement park, and parking lots (3.52 km straight‐distance from city center); (2) Desert Arboretum Park on the Arizona State University campus—mostly butte, desert‐scrub habitat (e.g., mesquite, palo verde, creosote) with nearby parking lots and athletics stadiums (13.56 km from city center); (3) Cholla Park—developed park with baseball field, tennis courts, playground, and some vegetation (28.09 km from city center); (4) Arizona Fruit Trees nursery—personal residence uniquely and fully vegetated with dozens of species of natural and artificial fruit trees, adjacent to largely xeriscaped homes in the neighborhood (39.39 km from city center).

Supporting information.

## Data Availability

The data that support the findings of this study are available from the corresponding author upon reasonable request.
