# Supplementary figures and images for: Variation in Plumage Coloration of Rosy‐Faced Lovebirds (Agapornis roseicollis): Links to Sex, Age, Nutritional Condition, Viral Infection, and Habitat Urbanization
Source: J Exp Zool A Ecol Integr Physiol. 2024 Sep 16;343(1):48–58. doi: 10.1002/jez.2867 (PMC11617812; doi:10.1002/jez.2867)

## Slide 1
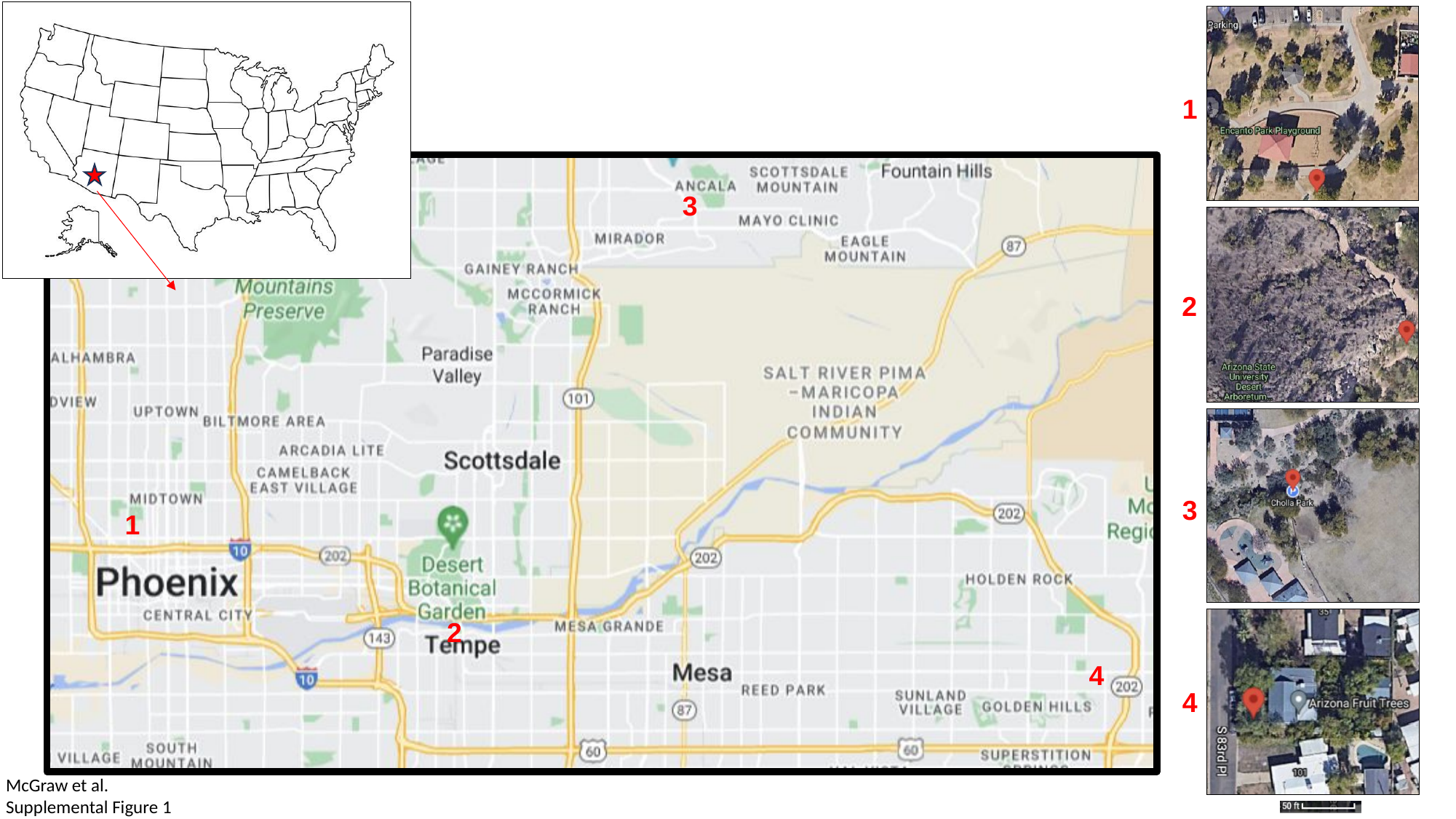

1
3
1
2
4
2
3
4
McGraw et al.
Supplemental Figure 1

Supplement: Supplementary file 1 — Supplemental Figure 1 Inset map (expanding local area from the red star demarcating the greater Phoenix, Arizona, USA metropolitan area) and satellite images of the 4 numbered study sites. (1) Encanto Park—developed park with artificial lakes, playground, walking paths, amusement park, and parking lots (3.52 km straight‐distance from city center); (2) Desert Arboretum Park on the Arizona State University campus—mostly butte, desert‐scrub habitat (e.g., mesquite, palo verde, creosote) with nearby parking lots and athletics stadiums (13.56 km from city center); (3) Cholla Park—developed park with baseball field, tennis courts, playground, and some vegetation (28.09 km from city center); (4) Arizona Fruit Trees nursery—personal residence uniquely and fully vegetated with dozens of species of natural and artificial fruit trees, adjacent to largely xeriscaped homes in the neighborhood (39.39 km from city center). [file JEZ-343-48-s001.pptx]
