# Supplementary material for: Variation in Plumage Coloration of Rosy‐Faced Lovebirds (Agapornis roseicollis): Links to Sex, Age, Nutritional Condition, Viral Infection, and Habitat Urbanization
Source: J Exp Zool A Ecol Integr Physiol. 2024 Sep 16;343(1):48–58. doi: 10.1002/jez.2867 (PMC11617812; doi:10.1002/jez.2867)
Supplement: Supplementary file 2 — Supporting information. [file JEZ-343-48-s002.docx]

Supplemental Table 1. Intercorrelations among response variables (plumage color metrics). Sat = saturation; bri = brightness; Tail = tail spot. Pearson correlation coefficients are shown; values in bold are statistically significant (p < 0.05).

|  | Face hue | Face sat | Face bri | Rump hue | Rump sat | Rump bri | Tail hue | Tail sat | Tail bri | Tail size |
| --- | --- | --- | --- | --- | --- | --- | --- | --- | --- | --- |
| Face hue | X | **-0.61** | -0.20 | 0.20 | -0.16 | 0.08 | -0.01 | -0.07 | -0.02 | -0.10 |
| Face sat | X | X | 0.20 | -0.01 | 0.20 | 0.01 | 0.10 | 0.11 | 0.07 | 0.05 |
| Face bri | X | X | X | 0.10 | 0.12 | -0.01 | 0.03 | 0.23 | -0.11 | 0.15 |
| Rump hue | X | X | X | X | **0.44** | 0.13 | -0.04 | -0.02 | 0.09 | -0.09 |
| Rump sat | X | X | X | X | X | -0.00 | -0.13 | 0.15 | 0.05 | -0.04 |
| Rump bri | X | X | X | X | X | X | 0.01 | -0.09 | 0.14 | 0.01 |
| Tail hue | X | X | X | X | X | X | X | **-0.48** | -0.04 | 0.02 |
| Tail sat | X | X | X | X | X | X | X | X | **0.28** | **0.31** |
| Tail bri | X | X | X | X | X | X | X | X | X | **0.56** |
| Tail size | X | X | X | X | X | X | X | X | X | X |

Supplemental Table 2. Intercorrelations among predictor variables. Pearson correlation coefficients are shown for comparisons of continuous variables, F-ratio values are shown for tests involving one categorial variable (e.g. age, sex, site, disease presence), and chi-square values are presented for comparisons with two categorial variables. Numbers in bold are statistically significant (p < 0.05).

|  | Age | Sex | Site | Condition | Glucose | Ketones | BFDV | BuFDV |
| --- | --- | --- | --- | --- | --- | --- | --- | --- |
| Age | X | χ^2^ = 0.05 | χ^2^ = 2.80 | F = 0.76 | F = 1.37 | F = 0.22 | χ^2^ = 0.80 | χ^2^ = 0.11 |
| Sex | X | X | χ^2^ = 2.69 | F = 0.01 | F = 0.20 | F = 0.19 | χ^2^ = 0.12 | χ^2^ = 3.29 |
| Site | X | X | X | F = 1.04 | F = 3.12 | F = 0.63 | χ^2^ = 0.16 | χ^2^ = 7.78 |
| Condition | X | X | X | X | r = 0.15 | **r = -0.33** | F = 0.16 | F = 0.15 |
| Glucose | X | X | X | X | X | r = 0.07 | F = 0.01 | F = 0.24 |
| Ketones | X | X | X | X | X | X | F = 0.00 | F = 0.04 |
| BFDV | X | X | X | X | X | X | X | χ^2^ = 0.65 |
| BuFDV | X | X | X | X | X | X | X | X |
